# Oral microbiome signatures of ageing and cognitive function across distinct geographical cohorts

**DOI:** 10.1371/journal.pone.0359559

**Published:** 2026-10-05

**Authors:** Joanna E. L’Heureux, Bart Roelands, Lee Wylie, Romain Meeusen, Andrew M. Jones, Anni Vanhatalo

**Affiliations:** 1 University of Exeter Medical School, Faculty of Health and Life Sciences, University of Exeter, St Luke’s Campus, Exeter, United Kingdom; 2 Human Physiology & Sports Physiotherapy Research Group Vrije Universiteit Brussel, Brussel, Belgium; 3 Laboratory of Sports and Nutrition Research, Riga Stradins University, Riga, Latvia; 4 Department of Sports, Recreation, Exercise and Sciences, Faculty of Community and Health Sciences, University of the Western Cape, Cape Town, South Africa; University of the Pacific, UNITED STATES OF AMERICA

## Abstract

Declining cognitive function in older adults may be linked to reduced nitric oxide (NO) bioavailability, which is supported by oral nitrate-reducing bacteria. Oral microbiome community structure varies geographically, complicating understanding of microbiome-cognition relationships across populations. This study aimed to identify consistent associations between the oral microbiome and cognitive function across two geographically distinct cohorts from the UK and Belgium, and to explore age-related differences in NO biomarkers, cognitive performance, and microbiome composition using 16S rRNA sequencing. Sixty healthy participants (15 young and 15 older adults per location) completed a cognitive test battery, blood pressure measurements, plasma and saliva collection for nitrate and nitrite analysis, and a tongue swab for oral microbiome assessment. Despite geographical differences in oral microbiome composition (PERMANOVA *P* = 0.04), age-related microbial shifts were consistent across geographic cohorts, including enrichment of *Treponema and Tannerella* in older adults. Nitrate-reducing taxa such as *Haemophilus parainfluenzae* and *Neisseria flavescens* were consistently associated with cognitive performance measures in both cohorts (*P* < 0.05). In the UK, higher plasma [nitrate] was associated with improved Digit Symbol Substitution Test (DSST) accuracy (β = 19.2, *P* < 0.01) and reaction times (β = −3444 ms, *P* < 0.05), but poorer Emotion Recognition Task (ERT) accuracy (β = −5.219, *P* < 0.05), while UK plasma [nitrite] was associated with slower Balloon Analog Risk Task (BART; β = 2277 ms, *P* < 0.001) and ERT (β = 8577 ms, *P* < 0.05) reaction times. In Belgium, saliva [nitrate] was associated with slower Visual Object Learning Test (VOLT) reaction times (β = 202 ms, *P* < 0.05), while in the UK, saliva [nitrite] correlated with slower BART (β = 111 ms, *P* < 0.05) and LOT (β = 1888 ms, *P* < 0.01). Overall, geography emerged as a major driver of oral microbiome variation, yet consistent age-related microbial and cognitive associations were preserved across populations. These findings emphasise the need to account for geographic context in microbiome-health research. A better understanding of oral microbial patterns may help inform future mechanistic or interventional studies to promote cognitive health in ageing.

## Introduction

A decline in the bioavailability of nitric oxide (NO) may significantly contribute to human neurodegeneration and cognitive deterioration during ageing, through downregulation of NO synthases. Following the consumption of food sources containing dietary nitrate such as beetroot juice, nitrate enters the blood circulation and is actively taken up by the salivary glands [1,2]. Commensal bacteria in the oral cavity reduce the concentrated salivary nitrate to nitrite, and some of the swallowed nitrite enters the bloodstream and increases plasma nitrite concentration [1]. The nitrite is further reduced to the signalling molecule and vasodilator, NO, under acidic or hypoxic conditions. Oral bacteria, therefore, play a key role in the nitrate-nitrite-NO pathway. Antimicrobial mouthwash use may reduce nitrate-reducing bacterial populations [3], and disrupt the nitrate-nitrite-NO pathway, thereby reducing NO bioavailability and attenuating the blood pressure lowering effects associated with inorganic dietary nitrate supplementation [4]. Recent evidence further suggested that over a two-week period, twice-daily use of an antiseptic mouthwash reduced oral microbial diversity and impaired flow-mediated dilation, compared to a high-nitrate dietary intervention [5]. Over 700 bacterial taxa have been identified in the oral cavity [6]. Disruptions to the oral cavity including long-term denture use, missing teeth, poor tooth hygiene and periodontal disease have been linked to mild cognitive impairment and neurodegenerative disease [7–9]. For instance, anaerobic bacteria such as *Porphyromonas gingivalis* and *Treponema denticola*, which are associated with chronic periodontitis, have been implicated in both cognitive decline and Alzheimer’s disease [10,11]. One study reported that individuals with oral disease experienced cognitive decline within six months of testing [12]. Human ageing has also been associated with changes in the oral microbiome, including a potential reduction in microbial diversity, which may contribute to altered oral and systemic health outcomes in older adults [13,14]. Therefore, understanding how the composition of nitrate-reducing oral bacteria changes throughout human ageing may provide a greater understanding of the relationship between NO bioavailability and cognitive function.

The composition of the human microbiome is widely recognised to vary according to geographical sampling location, climate zone, countries, and within different regions of the same country [15–17]. These differences are thought to be influenced by a range of factors, including host genetics, ethnicity, diet, environmental exposures, and lifestyle [18–21].

Although age-related changes in the oral microbiome and geographical variation in microbial composition have been described independently, it remains unclear whether age-related microbial patterns and their associations with cognitive performance are reproducible across geographically distinct populations. This gap is particularly relevant for understanding nitrate-reducing bacteria involved in the nitrate-nitrite-NO pathway, given their potential role in supporting vascular and cognitive function. Clarifying whether these microbial signatures of cognitive ageing are conserved across different environmental and lifestyle contexts, or instead reflect geographical location-specific patterns, is necessary for determining the generalisability of oral microbiome-cognition relationships.

In the present study, we aimed to determine whether consistent relationships between the oral microbiome and cognitive function exist across two geographical locations, the UK and Belgium, in young and older adults.

## Materials and methods

### Participants

The study was given ethical approval by the University of Exeter Ethics Committee (Sport and Health Sciences, ethical approval number 170209/B/03) and the Medical Ethical Committee UZ Brussels (BUN 143201630619). The participant recruitment period in the UK cohort was from 20^th^ of January 2017 until the 20^th^ of January 2018, and the participant recruitment period in the Belgium cohort was from 2^nd^ of August 2017 until December 2019. All participants provided their written informed consent before participating, and were eligible to participate in the study, passing the mini-mental state examination questionnaire and satisfying the eligibility criteria. There were 30 participants recruited from both Exeter (UK) and Brussels (Belgium) including 15 young and 15 older adults from both countries. The participants were screened before commencement of the study. All participants were in good health and were non-smokers. The participants reported that they did not regularly drink alcohol > 14% ABV or regularly use mouthwash. Participants were excluded if they currently or had recently used (within the past three months) antibiotics. Participants were also excluded if they had a history of or recent treatment for oral disease, cardiovascular disease, cancer, or upper respiratory tract infection. Participants were excluded from taking part if they currently used dietary supplements containing nitrate, L-arginine or L-citrulline. The participants were invited to complete questionnaires to assess their general health and suitability for the study, including an oral health assessment, a food frequency questionnaire and a physical activity questionnaire. The food frequency questionnaire data was assessed using FFQ EPIC Tool for Analysis [22]. The participants were asked to arrive at the laboratory fasted in the morning and to avoid caffeine and alcohol for 24 hours before the test.

### Study design

The participants visited the laboratory on two separate occasions. During the first visit, the participants completed health screening questionnaires and were familiarised with the cognitive tests (Joggle Research, Seattle, US). On the second visit, resting blood pressure and salivary flow rate were measured. A saliva sample was taken to assess the salivary concentrations of nitrate and nitrite, an oral swab was taken to sample the oral microbiome from the tongue dorsum, and a venous blood sample was taken to assess plasma nitrate and nitrite concentrations. The participants also completed the Joggle cognitive test battery. All experimental procedures were identical between the UK and Belgium sites, and all nitrate and nitrite analyses were performed using the same NO analyser based in the UK.

### Blood pressure

The participants were seated in a quiet room for 10 min prior to the measurement of blood pressure. Blood pressure of the brachial artery was measured using an automated sphygmomanometer (Dinamap Pro, GE Medical Systems, Tampa, USA). Four blood pressure measurements were taken with a 1-minute interval between each measurement. The mean systolic blood pressure (SBP), diastolic blood pressure (DBP) and mean arterial pressure (MAP) were calculated from the final three blood pressure measurements.

### Blood sampling

Resting venous blood samples were drawn by venepuncture from the antecubital fossa into lithium heparin blood sampling tubes and centrifuged at 4,000 rpm for 8 min at 4°C. The extracted plasma samples were stored at −80°C until the measurement of plasma nitrate and nitrite by ozone-based chemiluminescence.

### Measurement of plasma nitrate and nitrite by ozone-based chemiluminescence

The plasma samples were defrosted and 300 µL of plasma was deproteinised by adding 600 µL of ice-cold methanol. The samples were allowed to incubate for 30 min and centrifuged for 5 min at 10,000 g. For the analysis of nitrate, the water bath was set at 95°C and 100 µL of deproteinised plasma was injected into the NO analyser (Sievers NOA 280) purge vessel containing 0.1 M vanadium chloride (Sigma-Aldrich), with 1 M hydrochloric acid (Sigma-Aldrich), and 100 μl antifoam (Sigma-Aldrich). The sample concentrations were determined against standards of known sodium nitrate concentrations. For the analysis of plasma nitrite concentration, the water bath was set at 55°C and 100 µL of the deproteinised plasma was injected directly into the purge vessel containing 4.0 ml acetic acid (Sigma-Aldrich), 1.0 ml sodium iodide (Fisher Scientific), and 100 μl antifoam. The plasma nitrite concentrations were measured against a standard curve of known sodium nitrite (Fluka Analytical) concentrations.

### Saliva sampling and the measurement of salivary nitrate and nitrite by the modified Griess reaction

The samples for the determination of salivary nitrate and nitrite were collected by actively expectorating into a universal tube for 2-min intervals until approximately 1.5 mL of saliva was collected, and stored at −80°C. Salivary nitrate and nitrite concentration were measured by a validated altered Griess reaction plate method as described by Smallwood *et al,* [23]. The Griess reaction method has been evaluated against ozone-based chemiluminescence [24], and is effective for measuring the high nitrate and nitrite concentrations found in saliva.

### Oral microbiome sampling

The oral bacteria were collected by sweeping the middle of the tongue dorsum with medium pressure using a sterile buccal brush (QIAGEN Gentra Puregene Buccal Cell Kit). The oral swabs were immediately placed in a 1.5 mL microcentrifuge tube containing 600 µL of cell lysis solution (QIAGEN). The oral swabs were stored at −80°C until DNA extraction. The oral bacteria DNA extraction and sequencing preparation from the swabs was completed using the QIAGEN Gentra Puregene kit in accordance with the manufacturer’s instructions. Library preparation and PCR amplification of the 16S region was completed following the protocol in the BIOOScientific 16S v1-v3 amplicon seq kit. Following amplification, clean-up was completed using AMPure®XP beads (Becton Dickinson, Franklin Lakes, NJ) and indexing primers were added to the samples (Primer Index 141–200; BIOOScientific NEXTflex 16S V1-V3 Amplicon-Seq Barcoded Primers The oral microbiome library was sequenced using paired-end 300 bp reads on the Illumina MiSeq platform (Illumina, San Diego, CA).

### Oral microbiome bioinformatic processing

Initial quality control and adapter removal were performed using Trim Galore! (Babraham Bioinformatics, https://www.bioinformatics.babraham.ac.uk/projects/trim_galore/), following upstream preprocessing with fastp, which retained reads ≥75 base pairs. Raw paired-end 16S rRNA gene sequencing reads were processed using the DADA2 pipeline (v1.28) in R version 4.3.3 [25,26],. Forward and reverse reads were quality-filtered and trimmed, with truncation parameters selected based on inspection of per-base quality score profiles, followed by error model learning and denoising to infer amplicon sequence variants (ASVs). Paired reads were merged, and chimeras were removed. Taxonomic assignment was performed using the SILVA v138.1 reference database. The resulting ASV table, taxonomy, and sample metadata were combined into a Phyloseq object for downstream analysis [27]. Sequencing depth varied across samples (median 74,871 reads; range 8,081–278,274).

### Cognitive function tests

The participants were asked to complete a battery of cognitive tests in English or Dutch (Joggle Research Inc., Seattle, USA) on a portable electronic device (IPad Air, Apple Inc., USA). The battery included 10 validated cognitive tests that were selected to assess different cognitive abilities and areas of brain function [28,29]. The cognitive tests included memory, special orientation, abstract reasoning, visual tracking, risk decision making, sensor motor speed and attention. An overview of the cognitive test battery is shown in Table 1*.* A composite score was derived by standardising all outcome variables. Reaction time measures were first inverted to ensure that higher values reflected better performance. All reaction time, accuracy, and lapse variables were then normalised (z-scored), and the composite score was calculated as the unweighted mean of the cognitive metrics. Within each location and age group, the composite scores were evenly binned into low, average or high cognitive performers.

**Table 1:** Overview of the Joggle cognitive test battery.

Test	Assessed cognitive domain	Brain area recruited
Abstract Matching (AM)	Abstraction	Prefrontal Cortex
Balloon Analog Risk Task (BART)	Risk decision making	Orbital frontal cortex,
		Amygdala,
		Hippocampus,
		Anterior cingulate cortex
Digit Symbol Substitution Task (DSST)	Complex scanning, visual tracking	Temporal cortex,
		Prefrontal cortex,
		Motor cortex
Emotion Recognition Task (ERT)	Emotion identification	Cingulate,
		Amygdala,
		Hippocampus
Line Orientation Task (LOT)	Spatial orientation	Right temporo-parietal cortex,
		Visual cortex
Motor Praxis Task (MPT)	Sensory motor speed	Sensorimotor cortex,
Matrix Reasoning Task (MRT)	Abstract reasoning	Prefrontal cortex,
		Parietal cortex,
		Temporal cortex,
NBACK	Working memory	Dorsolateral prefrontal cortex,
		Cingulate,
		Hippocampus
Psychomotor Vigilance Test (PVT)	Vigilant attention	Prefrontal cortex,
		Motor cortex,
		Visual cortex,
Visual Object Learning Task (VOLT)	Visual learning, spatial working memory	Medial temporal cortex,
		Hippocampus

### Statistical methods

Taxa unclassified at the species level were removed. Species were filtered based on prevalence (≥5% of samples) and abundance (≥0.1% in at least one sample) and normalized to relative abundance. For downstream analysis, Hellinger-transformed species-level abundances were used where appropriate. Alpha diversity metrics were computed using the R vegan package [30]. Microbial community variation was explored using Principal Components Analysis (PCA) and Redundancy Analysis (RDA) and visualised using R microViz [31]. Species-level abundance data were Hellinger-transformed prior to ordination. Hellinger transformation was chosen to reduce the influence of highly abundant species and improves ordination of site differences. PCA was used to visualise unconstrained variation. Homogeneity of dispersions was confirmed (*P* > 0.05). Group differences in community composition were assessed using ADONIS2 PERMANOVA with 999 permutations in the R vegan package [30] based on Bray-Curtis dissimilarities. Bray-Curtis dissimilarity was selected because it effectively quantifies differences in community composition while remaining less sensitive to shared absences between samples [32]. To assess robustness, analyses were repeated using Aitchison distance derived from centered log-ratio (CLR) transformed abundances. RDA was applied to test how much variation could be explained by continuous variables, including age, sampling location, salivary and plasma nitrate and nitrite concentrations. Significance of RDA model terms and axes was assessed using permutation ANOVA (999 permutations). Differential abundance testing was performed using ANCOM-BC2, which estimates bias corrected log fold changes while accounting for compositionality and sampling fraction variation [33]. To assess whether country-associated differences persisted at the taxon level across age groups, the analysis included geographic location, age group, and their interaction as covariates. P‑values were adjusted using the Benjamini-Hochberg FDR procedure. To assess whether age moderated the relationship between plasma and salivary nitrate concentrations, nitrite concentrations, and cognitive performance, linear regression models were fitted separately within each geographical sampling location. Each model included a dependent variable cognitive outcome, and a nitrate or nitrite independent variable, with an interaction term between nitrate concentration, nitrite concentration, and age. P-values for interaction terms were FDR adjusted. For regression analysis of associations between microbial and physiological predictors and cognitive performance, the ten most influential ASVs from each location were selected based on principal component analysis (S1 Fig). Continuous predictors were scaled, and data were subset by geographical location (UK and Belgium). For each cognitive outcome, LASSO regression was applied using the glmnet R package [34] to identify a minimal set of predictive features. Features retained by LASSO were entered into multiple linear regression models. Predictors with zero variance or incomplete data were excluded.

## Results

### Participants

In the UK young adult group (n = 15), there were 11 males (mean ± SD; age, 23 ± 3 years; BMI, 26 ± 4) and 4 females (age, 23 ± 1 years; BMI, 25 ± 4). The UK older adult group (n = 15) included 6 males (age, 70 ± 8 years; BMI, 26 ± 2) and 9 females (age, 62 ± 8 years; BMI, 27 ± 5). In the Belgian young adult group (n = 15), there were 8 males (age, 26 ± 4 years; BMI, 25 ± 3) and 7 females (age, 24 ± 3 years; BMI, 21 ± 1). The Belgian older adult group (n = 15) included 6 males (age, 70 ± 4 years; BMI, 27 ± 2) and 9 females (age, 71 ± 4 years; BMI, 25 ± 3).

### Alpha diversity

There were no significant differences in Shannon diversity (H′) between geographical locations or age groups (*Kruskal-Wallis*, H = 4.4., *P* = 0.23; Fig 1A). There were also no significant differences in Chao1 species richness (H = 6.3, *P* = 0.1; Fig 1B). Similarly, when participants were grouped by composite cognitive performance, no significant differences were observed in either Shannon diversity (H = 3.3, *P* = 0.19; Fig 1C) or Chao1 richness (H = 0.46, *P* = 0.79; Fig 1D) across high, average, and low performance groups.

**Fig 1:**
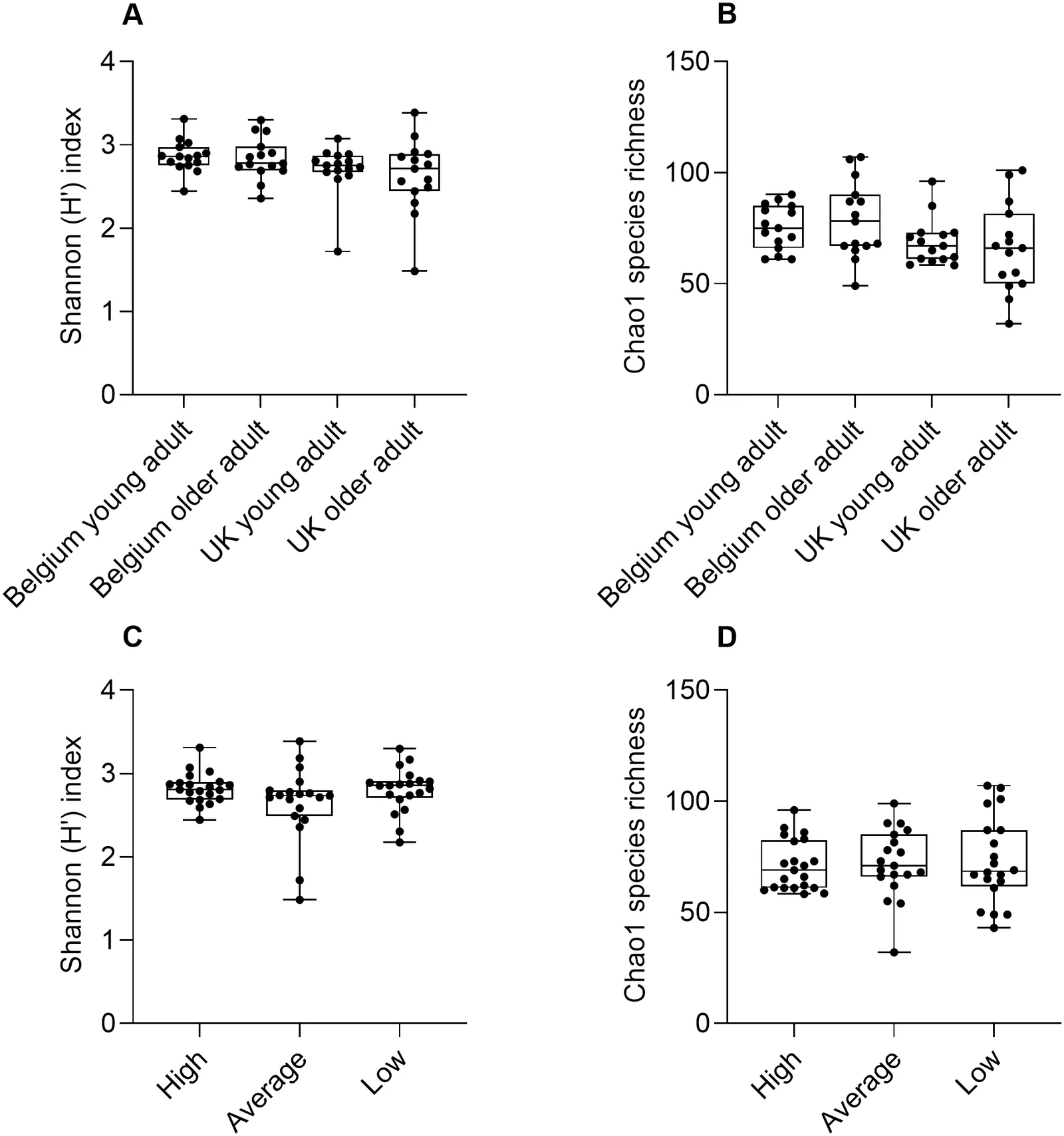
Alpha diversity metrics across participant groups. The boxplots show Shannon diversity index (A) and Chao1 species richness (B) stratified by sampling location (Young and older adults from Belgium and the UK. Boxplots also show Shannon diversity (C) and Chao1 richness (D) across composite cognitive performance groups (Low, Average, High). No significant differences in alpha diversity were observed across groups (Kruskal-Wallis, *P* > 0.05).

### Ordination

The first two PCA principal components explained 10.0% and 7.8% of the total variance, respectively. Taxa contributing most strongly to PC1 included *Neisseria subflava*, *Prevotella histicola*, and *Veillonella atypica*. PERMANOVA on Bray-Curtis dissimilarities indicated a significant difference in overall microbial community composition between age and location groups (Belgium young adults, Belgium older adults, UK young adults, UK older adults; R² = 0.08, F = 1.63, *P* = 0.04; Fig 2A). Pairwise comparisons revealed no significant differences between groups after FDR correction. RDA determined the influence of six continuous variables (age, geographical location, plasma and salivary nitrate and nitrite concentrations). The full RDA model explained 15.8% of the total variance in community composition and was statistically significant (R² = 0.16, F = 1.35, *P* = 0.05; Fig 2B). Among individual predictors, only geographical location contributed significantly to explaining microbial variation (F = 2.54, *P* = 0.01), while other variables did not reach significance (*P* > 0.1). None of the individual RDA axes were significant in permutation tests (*P* > 0.2), suggesting that the explanatory power is shared across multiple components.

**Fig 2:**
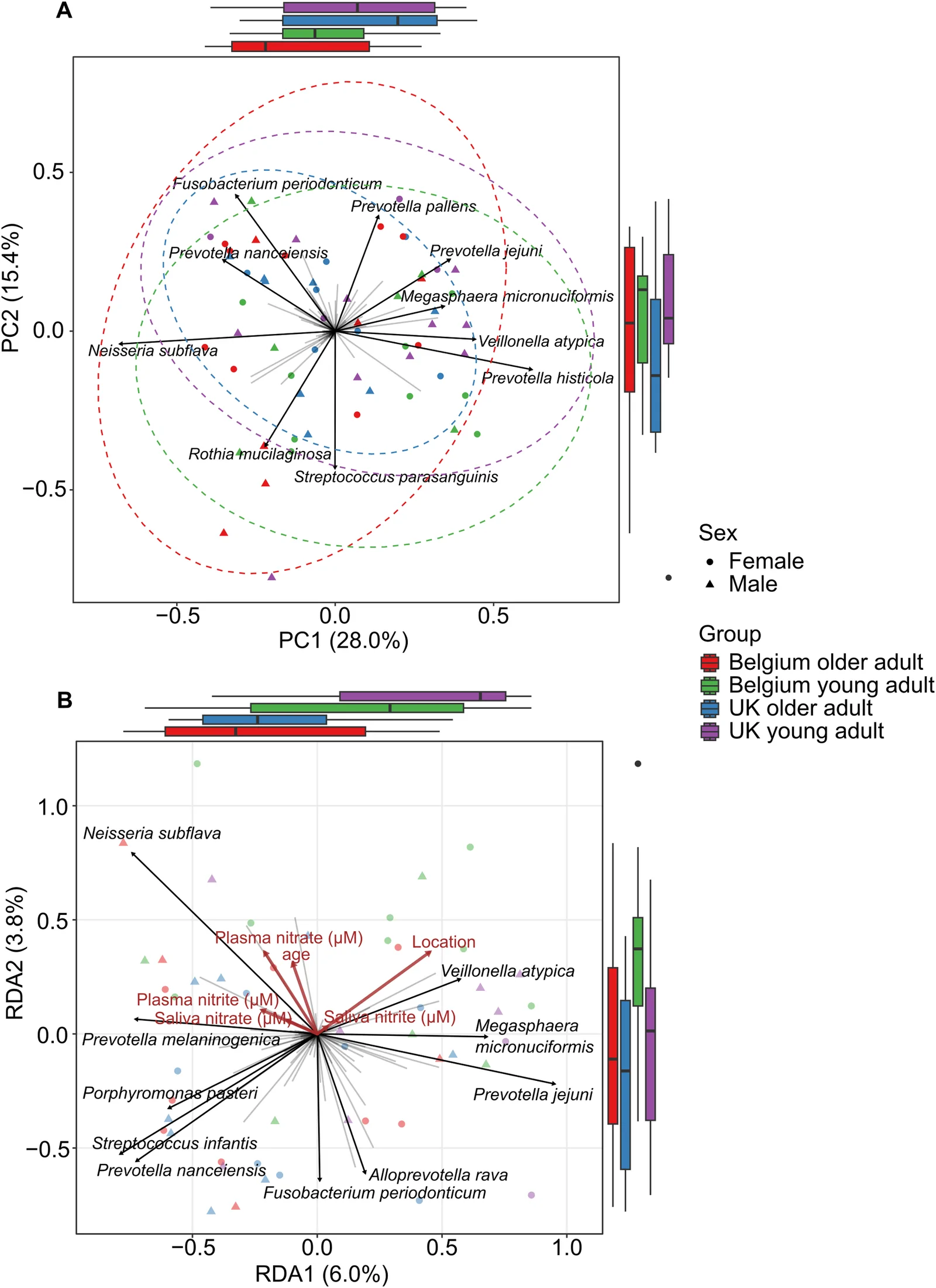
Ordination of oral microbiome communities. Principal Component Analysis (A) of oral microbiota shows separation by participant group (red: Belgium older adult, green: Belgium young adult, blue: UK older adult, purple: UK young adult). Group differences were significant (*P* = 0.04, PERMANOVA). PC1 and PC2 explain 10.0% and 7.8% of the variance, respectively. Redundancy Analysis (B) constrained by age, geographical location, and plasma and saliva nitrate and nitrite. The full model was significant (*P* = 0.05), with sampling location the only significant term (*P* = 0.01). Red arrows indicate explanatory (constraint) variables; black arrows represent microbial taxa contributing to ordination patterns. In both panels, boxplots display the distribution of samples along each axis by group.

### Analysis of consistent age-related taxa between geographic locations

ANCOM-BC2 identified 17 salivary bacterial species with significant differences in estimated relative abundance between younger and older adults (q < 0.05 for the age effect), with no evidence that the effect of age differed between countries (q > 0.05), indicating consistent age-associated changes across Belgium and the UK (Fig 3A). Among these, eight species were enriched in older adults, including *Prevotella fusca* (log fold change [LFC]=2.44, *q* = 0.01), *Actinomyces bouchesdurhonensis* (LFC = 1.52, *q* = 0.03), *Filifactor alocis* (LFC = 1.02, *q* = 0.02), *Leptotrichia buccalis* (LFC = 0.97, *q* = 0.01), *Treponema medium* (LFC = 0.95, *q* = 0.01), *Corynebacterium matruchotii* (LFC = 0.92, *q* = 0.02), *Aggregatibacter segnis* (LFC = 0.92, *q* = 0.03), and *Treponema amylovorum* (LFC = 0.89, *q* = 0.04). In contrast, nine species were enriched in younger adults, including *Prevotella scopos* (LFC = −2.18, *q* = 0.001), *Oribacterium asaccharolyticum* (LFC = −1.46, *q* = 0.002), *Fusobacterium hwasookii* (LFC = −1.39, *q* = 0.02), *Neisseria flavescens* (LFC = −1.33, *q* = 0.02), *Peptostreptococcus stomatis* (LFC = −1.32, *q* = 0.03), *Solobacterium moorei* (LFC = −1.23, *q* = 0.03), *Cryptobacterium curtum* (LFC = −1.23, *q* = 0.02), *Oribacterium sinus* (LFC = −1.14, q = 0.03), and *Actinomyces pacaensis* (LFC = −0.98, *q* = 0.04). At the genus level, ANCOM-BC2 identified two genera with significant age-associated differences (Fig 3B) and both genera were enriched in older adults, which included *Tannerella* (LFC = 1.79, *q* = 0.004) and *Treponema* (LFC = 1.65, *q* = 0.04).

**Fig 3:**
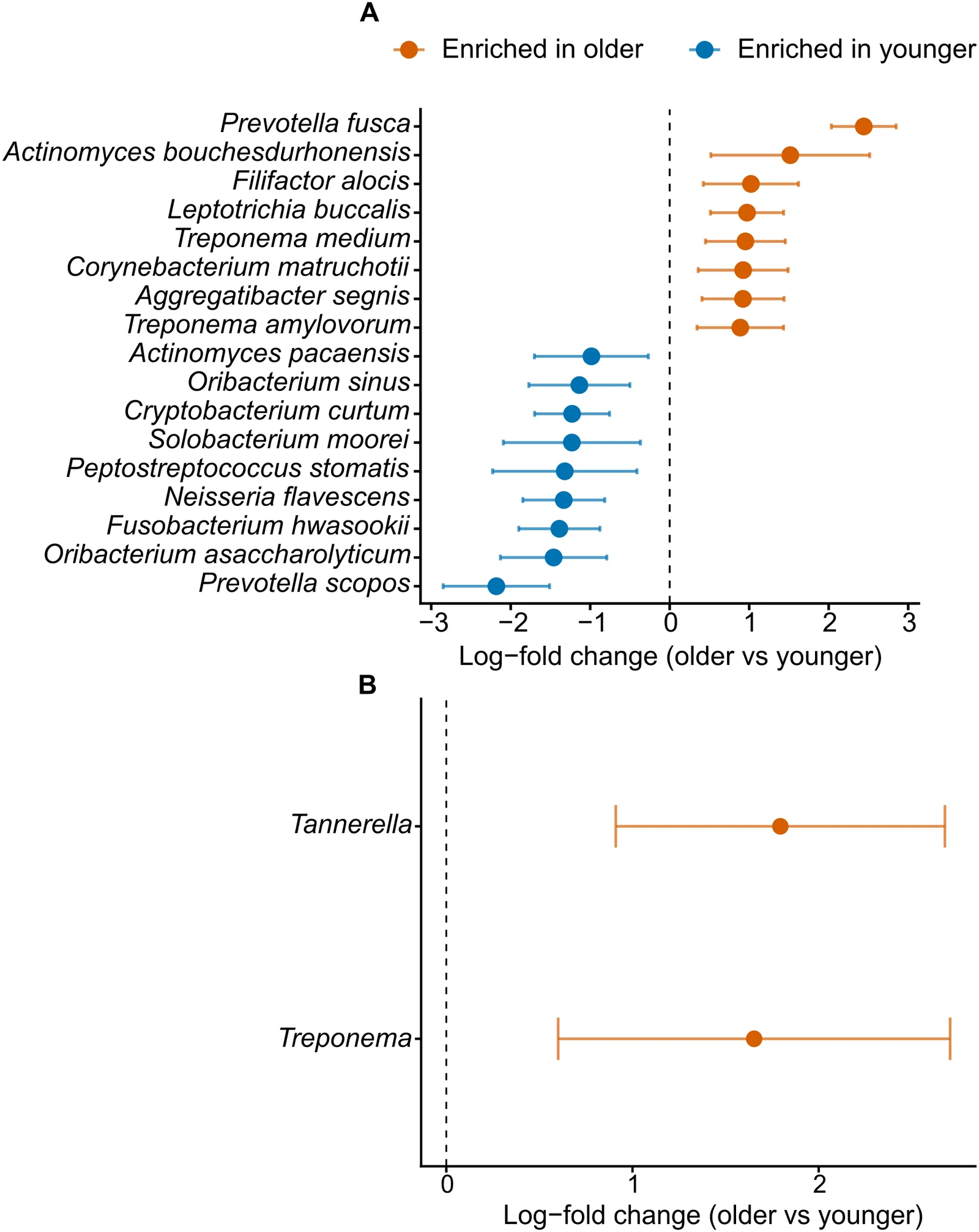
Age-associated differences in the oral microbiome across Belgium and the UK. ANCOM-BC2 identified oral bacteria associated with age in tongue swab samples from Belgium and the UK, with no evidence that the effect of age differed between countries (q > 0.05). (A) Species, and (B) genus-level showing significant differences in estimated relative abundance between younger and older adults (q < 0.05). Positive log values indicate enrichment in older adults; negative values indicate enrichment in younger adults. Taxa are ordered by effect size.

### Regression analysis of oral microbiome, physiological and cognitive associations

Age was consistently and strongly associated with cognitive performance across both populations. In Belgium, increasing age was associated with a lower composite score (β = −0.09 per year, *P* < 0.001), indicating a small but significant decline. Similarly, age was linked to poorer episodic memory (AM correct, β = −1.66, *P* < 0.01) and processing speed (DSST correct, β = −12.47, *P* < 0.001), with older individuals showing slower reaction times (DSST RT, β = 220 ms, *P* < 0.001). Age was also associated with slower responses on the MPT (β = 118 ms, *P* < 0.01) and VOLT (β = −1.04 correct, *P* < 0.01), as well as lower NBACK accuracy (β = −3.76, *P* < 0.001) and MRT accuracy (β = −1.78, *P* < 0.001). Comparable associations were observed in the UK cohort, where age negatively impacted DSST correct (β = −12.81, *P* < 0.001) and composite score (β = −0.08, *P* < 0.001), and was associated with slower cognitive reaction times across multiple tests including DSST (β = 264 ms, *P* < 0.001), MPT (β = 100 ms, *P* < 0.001), MRT (β = 1970 ms, *P* < 0.01), and PVT (β = 39 ms, *P* < 0.001). Age also predicted reduced NBACK performance (β = −2.60, *P* < 0.01), increased PVT lapses (β = 2.91, *P* < 0.01), and lower VOLT accuracy (β = −0.77, *P* < 0.05).

Microbial taxa showed both consistent and location-specific associations with cognitive measures. In Belgium, *Haemophilus parainfluenzae* was linked to faster reaction times on the ERT (β = −783 ms, *P* < 0.01) and VOLT (β = −520 ms, *P* < 0.001) tasks, as well as higher accuracy on the NBACK task (β = 1.90, *P* < 0.05). In contrast, in the UK cohort, *Haemophilus parainfluenzae* was associated with slower ERT reaction time (β = 394 ms, *P* < 0.05) but faster reaction times on the AM (β = −619 ms, *P* < 0.05) and LOT (β = −1269 ms, *P* < 0.01) tasks. Other notable associations include *Prevotella histicola*, which was associated with faster AM reaction times in the UK (β = −448 ms, *P* < 0.01) and improved VOLT reaction times in Belgium (β = −434 ms, *P* < 0.01). *Fusobacterium periodonticum* was linked to poorer ERT accuracy in the UK (β = −2.39, *P* < 0.001), while Prevotella jejuni was associated with slower ERT reaction times in the UK (β = 354 ms, *P* < 0.01). In Belgium, *Alloprevotella rava* was associated with better ERT performance (β = 1.52, *P* < 0.05) but slower VOLT reaction times (β = 299 ms, *P* < 0.01). *Neisseria subflava* was also associated with slower VOLT reaction times in Belgium (β = 393 ms, *P* < 0.001). Meanwhile, in the UK, *Prevotella nanceiensis* correlated with increased lapses on the PVT task (β = −2.95, *P* < 0.05). Some taxa exhibited location-specific effects. For example, *Veillonella atypica* was linked to reduced accuracy (VOLT correct, β = −1.27, *P* < 0.01) and slower reaction times (VOLT RT, β = 265 ms, *P* < 0.05) only in Belgium.

Mean arterial pressure (MAP) showed consistent associations with slower cognitive response times. In Belgium, MAP was associated with slower RTs on the AM (β = 513 ms, *P* < 0.05), ERT (β = 705 ms, *P* < 0.05), and VOLT tasks (β = 265 ms, P < 0.05). In the UK, higher MAP was associated with longer AM RT (β = 598 ms, *P* < 0.01), LOT RT (β = 1448 ms, *P* < 0.001), and PVT RT (β = 33 ms, *P* < 0.001), along with more PVT lapses (β = −3.05, *P* < 0.001). Additional physiological variables showed significant associations. In the UK, higher BMI was associated with better ERT accuracy (β = 2.28, *P* < 0.001), whereas in Belgium, BMI was negatively associated with LOT accuracy (β = −0.90, *P* < 0.05) and was associated with slower VOLT RT (β = 332 ms, *P* < 0.01). Plasma nitrate in the UK was associated with better DSST performance (correct: β = 19.2, *P* < 0.01; RT: β = −344 ms, *P* < 0.05) but poorer ERT accuracy (β = −5.19, *P* < 0.05). Plasma nitrite was associated with slower RTs in the UK on BART (β = 227 ms, *P* < 0.001) and ERT (β = 857 ms, *P* < 0.05). In Belgium, saliva nitrate was associated with slower VOLT RT (β = 202 ms, *P* < 0.05), and in the UK, saliva nitrite was linked to slower RTs on BART (β = 111 ms, *P* < 0.05) and LOT (β = 1888 ms, *P* < 0.01; Fig 4).

**Fig 4:**
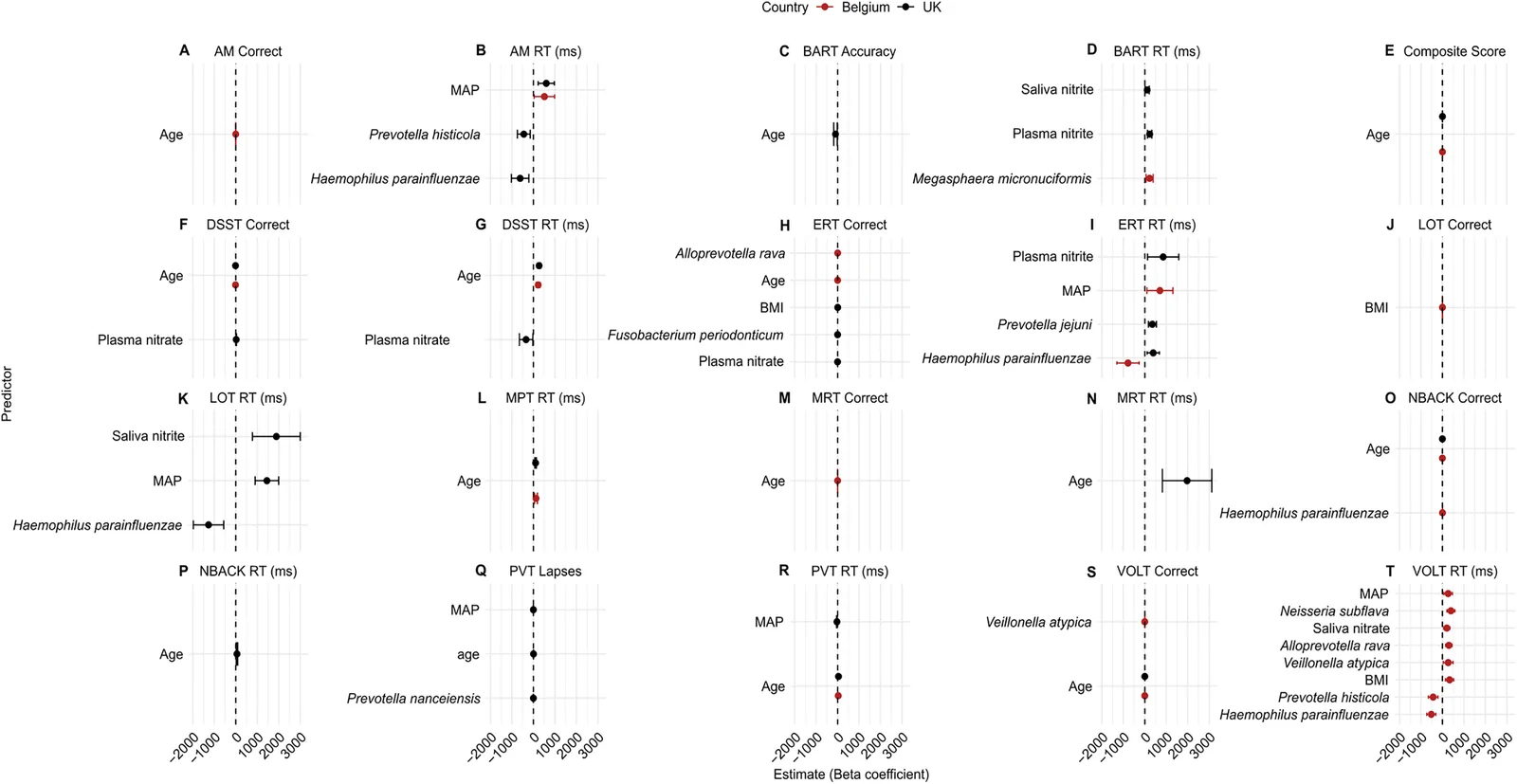
Associations between oral microbiota, physiological variables, and cognitive performance in UK and Belgium cohorts. Multiple linear regression analyses show significant relationships between specific oral bacterial species, physiological measures (age, BMI, MAP, plasma and saliva nitrate and nitrite concentrations), and cognitive test outcomes across two populations. Plots show standardised regression coefficients (β) for taxa and covariates significantly associated with cognitive scores (*P* < 0.05). Red coefficients show Belgium, black shows the UK. Panels A-T correspond to individual cognitive outcomes as indicated. Abbreviations: AM, abstract matching; BART, balloon analogue risk task; Correct, correct answers; DSST, digit symbol substitution test; ERT, emotion recognition task; LOT, line orientation task; MPT, motor praxis task; MRT, matrix reasoning task; NBACK, n-back task; PVT, psychomotor vigilance task; VOLT, visual object learning task; RT, reaction time; BMI, body mass index; MAP, mean arterial pressure.

### Plasma and saliva nitrate and nitrite concentrations

A two-way ANOVA on plasma nitrate concentrations showed no significant interaction between age and location (F = 1.69, *P* = 0.20), but there was a significant main effect of location (F = 4.35, *P* = 0.04). No significant main effect of age was found (F = 1.86, *P* = 0.18). Post hoc analysis showed higher nitrate concentrations in UK compared to Belgium among young adults (mean difference = 69.20 µM, *P* = 0.02). No significant differences were observed between age groups within either country or between countries among older adults (Fig 5A). Two-way ANOVA of plasma nitrite showed a significant interaction between age and location (F = 21.07, *P* < 0.0001). There were no significant main effects of age (F = 0.21, *P* = 0.65) or location (F = 0.53, *P* = 0.47). Post hoc tests showed that in Belgium, young adults had significantly higher plasma nitrite concentration compared to older adults (mean difference 0.49 µM, *P* = 0.005), whereas in the UK, older adults had higher concentrations than young adults (mean difference 0.60 µM, *P* < 0.001). Additionally, young adults in Belgium had higher plasma nitrite concentrations compared to those in the UK (mean difference 0.64 µM, *P* < 0.001), while older adults in Belgium had lower concentrations than their UK counterparts (mean difference 0.46 µM, *P* = 0.009; Fig 5B). There were no significant effects of age (F = 0.92, *P* = 0.34), location (F = 0.26, *P* = 0.61), or their interaction (F = 0.42, *P* = 0.52). Post hoc comparisons showed no significant differences between age groups within Belgium or the UK, nor between countries within either age group (*P* > 0.05, Fig 5C). For salivary nitrite, there was a significant effect of age (F = 4.25, *P* = 0.04), with young adults having higher levels than older adults (mean difference = 148.8 µM). No significant effects were found for location (F = 2.67, *P* = 0.11) or the interaction between age and location (F = 2.23, *P* = 0.14). Post hoc comparisons showed that UK young adults had significantly higher nitrite levels than both UK older adults (mean difference = 256.6 µM, *P* = 0.02) and Belgian young adults (mean difference = 225.8 µM, *P* = 0.03), while no significant differences were found between age groups in Belgium or between countries among older adults (Fig 5D).

**Fig 5:**
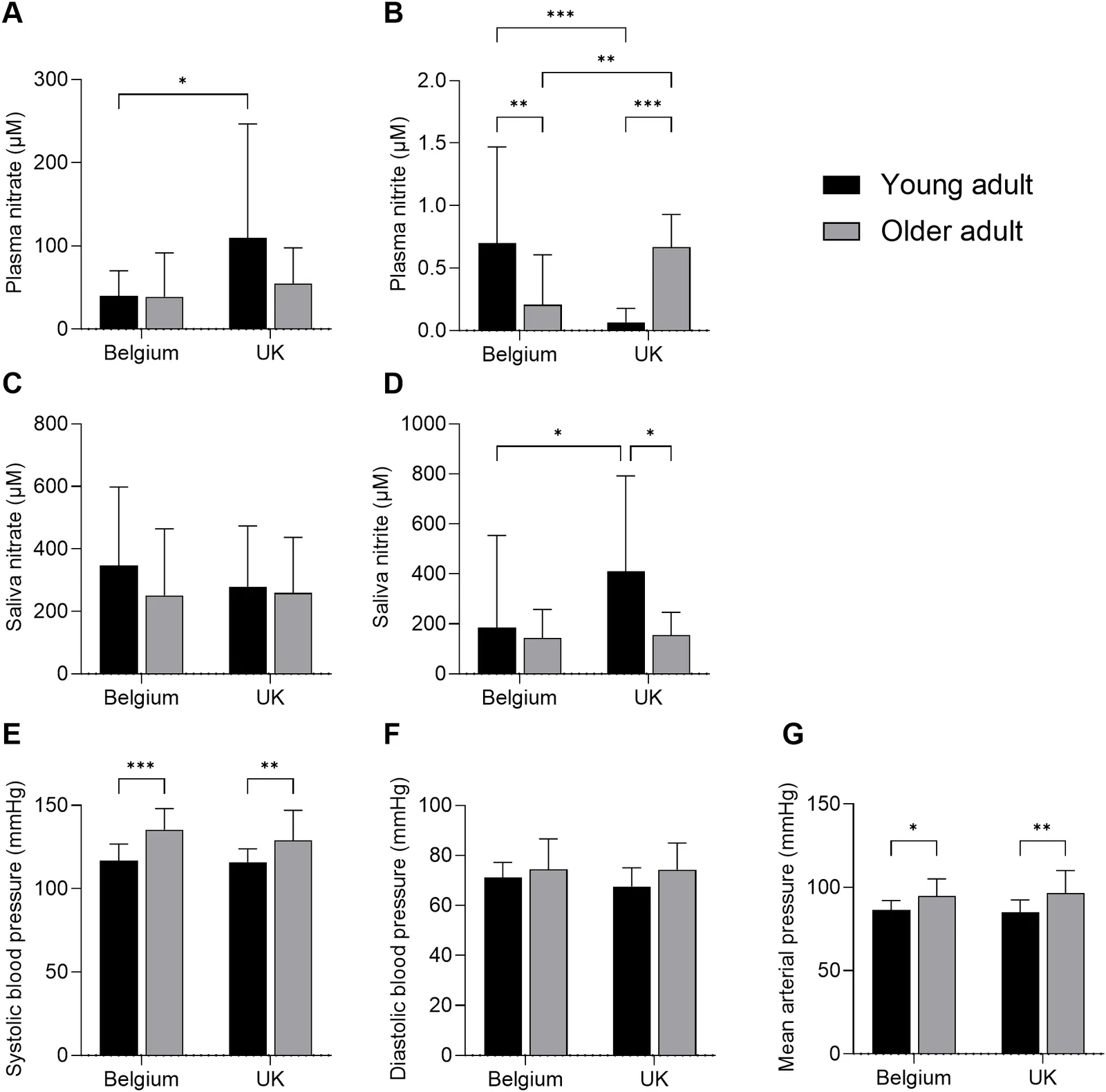
Group comparisons of plasma and saliva nitrate and nitrite concentrations and blood pressure by age group and geographical location. Bar plots illustrate differences between young and older adults across Belgium and UK sampling locations for (A) plasma nitrate, (B) plasma nitrite, (C) saliva nitrate, (D) saliva nitrite, (E) systolic blood pressure (SBP), (F) diastolic blood pressure (DBP), and (G) mean arterial pressure (MAP). Data are shown as mean ± standard deviation, *P < 0.05, **P < 0.01, ***P < 0.001.

### Blood pressure

Older adults had significantly higher SBP, DBP, and MAP compared to young adults. There was a strong age effect on SBP (F = 23.5, *P* < 0.001), with no effects of location (F = 1.22, *P* = 0.27) or age and location interaction (F = 0.73, *P* = 0.40). Post hoc comparisons showed that older adults had significantly higher SBP than young adults by 19 mmHg in Belgium (*P* = 0.0002) and by 13 mmHg in the UK (*P* = 0.007), with no differences between countries within age groups (Fig 5E). Age was significant for DBP (F = 4.15, *P* = 0.05), with no significant effects of location (F = 0.56, *P* = 0.46) or interaction (F = 0.54, *P* = 0.47). None of the post hoc comparisons for DBP reached significance (*P* > 0.05, Fig 5F). Similarly, MAP was elevated in older adults (F = 15.8, *P* < 0.001), with no effect of location (F = 0.004, *P* = 0.95) or interaction (F = 0.40, *P* = 0.53). Post hoc analyses showed significantly higher MAP in older adults compared to young in both Belgium (8 mmHg, *P* = 0.02) and the UK (11 mmHg, *P* = 0.002), with no differences between countries for either age group (Fig 5G).

### Age-dependent effects of plasma and salivary nitrate and nitrite on cognition

After correcting for multiple comparisons within each location, no interaction effects reached statistical significance (FDR-adjusted *P* > 0.05). Within each location, age did not significantly modify the relationship between plasma and saliva nitrate/nitrite concentrations and cognitive performance tests (S1 Table).

## Discussion

In the present study, we identified consistent associations between the oral microbiome, NO biomarkers, and cognitive function across two geographical locations, despite global differences in oral bacteria composition.

### Oral microbiome composition differed by geographic location

While no significant differences were observed in alpha diversity metrics across geographical location or age groups, we did detect significant variation in overall oral microbiome composition across age and location groups. In the RDA, geographical location emerged as the only significant predictor of microbial variation. Together, these findings align with previous research, suggesting that geographical location plays an important role in shaping the oral microbiome [35]. This may be due to various factors, including distance from the equator, dietary practices, cultural influences, socio-economic factors, and dental care [35–37]. PCA identified that several taxa contributed strongly to the observed variation. The species are commonly found in the human oral microbiota and have been associated with key functional roles. *Neisseria subflava* and *Veillonella atypica* have been implicated in oral nitrate reduction [38,39], while *Haemophilus parainfluenzae* may interact with mucosal immune pathways [40]. *Prevotella histicola* and *Fusobacterium periodonticum* have been linked to oral inflammation, periodontal and systemic disease [41,42]. The prominent influence of these species in the PCA of the full cohort suggests that these taxa may be ecologically important drivers of inter-individual, or group-level variation in oral microbiome composition.

### Taxa enriched in older adults show signatures of periodontal-associated communities

ANCOM-BC2 revealed age-related changes in the oral microbiome, in agreement with previous studies [43,44]. Our study provides further evidence of consistent age-related microbial changes in the oral microbiome across two geographically distinct cohorts in Belgium and the UK. Several taxa that were enriched in older adults are characteristic members of mature plaque and periodontal associated communities, including species *Filifactor alocis*, *Leptotrichia buccalis*, and *Aggregatibacter segnis* [45–47]. *Tannerella forsythia* and *Treponema denticola* which were enriched in older adults, form part of the “red complex” consortium which has been associated with both chronic periodontitis and cardiovascular disease [48]. Enrichment of periodontal disease-associated oral bacteria in older adults in both Belgium and the UK is consistent with the development of an inflammatory oral environment often observed in ageing, known as ‘inflammaging’ [49].

Importantly, periodontal pathogens have increasingly been implicated in cognitive decline [50,51]. For example, *Treponema* species may have increased abundance in elderly adults with cognitive impairment [52], and *Treponema* denticola has been reported in studies examining microbial signatures in Alzheimer’s disease [53]. Similarly, the oral pathogen, *Tannerella forsythia,* has been associated with elevated systemic inflammatory markers [54], and with reduced systemic NO bioavailability in experimental infected mouse models [55]. This suggests a possible association between periodontal dysbiosis and systemic NO metabolism.

*Neisseria flavescens* was significantly more abundant in younger adults than in older adults, and this age-associated difference was consistent across countries. *Neisseria* species have been observed in several studies to increase with dietary nitrate supplementation and are associated with lower oral disease and better cardiovascular health outcomes [56–58]. A higher abundance of *Neisseria flavescens* in younger adults is consistent with previous research suggesting a more favourable oral environment for nitrate metabolism during early adulthood [43], and may be relevant for understanding responses to inorganic dietary nitrate supplementation. Longitudinal and mechanistic studies are required to determine whether conserved microbial signatures contribute to ageing biology or primarily reflect downstream consequences of changes in host physiology.

### Differentially abundant taxa were identified between cognitive performance groups within each country, but no consistent patterns emerged across locations

We performed differential abundance analysis within each geographical location, stratified by cognitive performance group. Although the associations were country-specific, a common pattern emerged across both cohorts, whereby lower cognitive performance was associated with enrichment of taxa linked to oral inflammation or dysbiosis, such as *Treponema* and *Prevotella veroralis* [42,53]. This aligns with literature suggesting that chronic oral inflammation may influence systemic vascular and immune pathways implicated in cognitive decline [59]. Interestingly, *Neisseria flavescens* was significantly enriched in the lower-performing group within the UK cohort, contrasting with our previous findings showing that Neisseria at the genus level was associated with positive cognitive outcomes in older adults [51]. *Neisseria* species have been considered potentially beneficial due to their role in nitrate metabolism and maintenance of oral health [57,58]. This enrichment may reflect subtle changes in oral ecological balance or compensatory microbial dynamics. It is important to note that all participants in this study were healthy, and cognitive performance was stratified relative to the cohort within each location. Therefore, differences reflect subclinical variation rather than cognitive impairment. While differentially abundant taxa between cognitive performance groups were identified, there were no consistent outcomes between geographical locations.

Several associations between microbial taxa, physiological variables, and cognitive outcomes differed in direction across cognitive domains, cohorts, and individuals. These directional differences are not unexpected, because cognitive tasks show substantial inter-individual variability [60]. Furthermore, the cognitive tasks assessed distinct neurocognitive functions [61] such that microbial associations may vary by cognitive domain and may not exhibit uniform patterns across all tasks or cohorts. For example, *Haemophilus parainfluenzae* was linked to faster reaction times on several tasks in Belgium but showed both positive and negative associations across tasks in the UK, reflecting domain-specific rather than global cognitive effects. The observed associations should therefore be considered to be exploratory, and larger studies will be required to determine whether these patterns reflect true biological differences or statistical variability. Future work may also benefit from grouping individual cognitive tests into broader cognitive domains (e.g., processing speed, working memory, visuospatial reasoning), rather than analysing each test separately.

### Consistent microbial and physiological predictors of cognitive function across populations

Age emerged as the most consistent and robust predictor of cognitive performance across both Belgium and UK cohorts. In both populations, increasing age was associated with lower composite cognitive scores, reduced accuracy, and slower reaction times across multiple domains, including episodic memory, attention, working memory, and processing speed. These findings align with well-established evidence showing age-related declines in cognitive function and underscore the reproducibility of these associations in geographically distinct samples [62]. Beyond age, microbial taxa showed both shared and region-specific associations with cognition. In both cohorts, *Prevotella histicola* consistently correlated with faster reaction times. Interestingly, while *Prevotella histicola* has been associated with dental caries in the oral cavity [42], the species has also been linked to short-chain fatty acid production in the gut, which has been associated with cognitive function through the gut-brain axis [63,64]. Further studies are needed to determine whether oral and gut strains of *Prevotella histicola* are linked and to clarify how this connection may impact cognitive function.

In both cohorts, *Haemophilus parainfluenzae* was significantly associated with cognitive performance, though the specific tests and direction of these associations differed. In Belgium, higher abundance of *H. parainfluenzae* was associated with faster reaction times on the ERT and VOLT tasks, as well as improved accuracy on the NBACK task, indicating a potential beneficial association with cognitive processing speed and accuracy. Conversely, in the UK cohort, *Haemophilus parainfluenzae* was associated with slower ERT reaction times but faster reaction times on the AM and LOT tasks. We previously identified modular correlations between *Haemophilus parainfluenzae* and *Neisseria*, together linking these taxa to cognitive function in older adults [51]. Additionally, *Haemophilus* spp. have been implicated in contributing to the nitrate-nitrite-NO pathway [65]. These findings further support the role of specific oral bacteria, such as *Haemophilus parainfluenzae*, in associating with cognitive performance. Several other taxa showed location-specific associations, but collectively, the results highlight that certain bacteria repeatedly associate with faster cognitive responses and higher accuracy, particularly those enriched in younger adults and involved in the oral nitrate-nitrite-NO pathway.

### Physiological associations with cognitive performance across populations and age groups

Our results revealed distinct associations between plasma and saliva nitrate and nitrite concentrations and cognitive performance in the UK and Belgium cohorts. In the UK, higher plasma nitrate was linked to better DSST accuracy and faster reaction times but poorer ERT accuracy, indicating task-specific effects. Plasma nitrite was associated with slower reaction times on BART and ERT. In Belgium, saliva nitrate was associated with slower VOLT reaction times, while in the UK, saliva nitrite correlated with slower responses on BART and LOT. Supplementation with inorganic dietary nitrate may enhance cognitive performance, likely mediated through increasing NO bioavailability [66,67]. However, our participants were not supplemented, and resting nitrate and nitrite levels showed mixed patterns. Therefore, we would not expect strong associations between fasted, baseline circulatory nitrate and nitrite concentrations and cognitive performance. Plasma nitrate concentration was higher in UK young adults compared to their Belgian counterparts, with no significant differences between age groups. In contrast, plasma nitrite showed a significant interaction between age and location. Belgian young adults had higher nitrite concentrations than both Belgian older adults and UK young adults, while UK older adults exhibited higher nitrite concentrations than UK young adults. Although age-related decreases in plasma nitrite have been reported previously [68,69], this finding was only consistent in the Belgian cohort. Conversely, a previous UK-based study found no significant age-related differences in baseline plasma nitrite, with a tendency toward higher concentrations in older adults [70]. These mixed findings may reflect geographic differences in diet, lifestyle, or oral microbiome activity.

A diet high in inorganic dietary nitrate will significantly impact plasma and saliva nitrate and nitrite concentrations [71]. This study is limited by the lack of dietary control, which likely contributed significant variability to baseline nitrate and nitrite levels, complicating the interpretation of their associations with cognition and the oral microbiome. As expected, SBP and MAP were significantly lower in young adults compared to older adults in both geographical locations, as shown previously [72]. Increased MAP was strongly linked to slower reaction times on multiple tasks in both Belgium and the UK. In a previous study, increased MAP was correlated with low cerebral blood flow, which could impact cognitive performance, particularly in older adults [73] highlighting the contribution of vascular health to cognitive performance. The increased blood pressure during ageing may be a result of arterial stiffness and impaired NO bioavailability [74], as NO is a major contributor to blood pressure homeostasis.

### Methodological considerations

The oral cavity harbours multiple distinct microbial community niches, and community composition differs substantially between oral sites [75]. In the present study, the tongue dorsum was selected for microbiome sampling because it has been found to be a major site of oral nitrate reduction, with a previous study demonstrating that a large proportion of oral nitrate-reducing activity occurs on the tongue dorsum [76]. In our previous work, we found that while saliva samples contained a higher proportion of nitrate-reducing bacteria compared to the tongue dorsum, saliva samples had greater intra-individual variation in bacteria abundance [77]. As this study compared younger and older adults, age-related differences in salivary bacterial composition may partly reflect differences in salivary flow rate. It is known that salivary flow rate is an important determinant of oral microbial composition [78] and can vary across sampling days. Moreover, salivary flow rate tends to decline with age, with older adults typically exhibiting lower flow rates than younger adults [79]. Therefore, to overcome this potential confounder, the tongue dorsum was selected as an appropriate oral microbiome sampling location.

The Belgian samples were transported in liquid nitrogen to the UK for analysis. Therefore, there were storage inconsistencies between the UK and Belgian samples; however, although these differences may have introduced some variability, we would not anticipate this to systematically influence the nitrate and nitrite measurements. It was beyond the scope of this study to assess a more comprehensive range of variables that may affect cognition, NO bioavailability or the oral microbiome, including factors such as habitual diet, physical activity, and sleep deprivation. A diet high in inorganic dietary nitrate may impact oral bacteria community composition, favouring nitrate-reducing bacteria [80]. Further research should include more a comprehensive assessment of participant characteristics and consider collecting samples on multiple days to mitigate day to day variability in outcome variables. Despite these methodological considerations, significant age and location-related differences were still observed, suggesting that the main findings were robust.

Finally, it is acknowledged that the sample size in this study was relatively small such that the findings should be considered to be exploratory and associative rather than confirmatory. Larger studies are required to validate these findings, assess their robustness, and determine whether the observed associations generalise to broader populations.

## Conclusions

This study underscores the critical influence of geographical location in shaping oral microbiome composition, which must be carefully considered when interpreting microbiome–health relationships and designing targeted interventions. Despite differences in oral bacterial communities and nitrate/nitrite biomarker profiles between the Belgian and UK cohorts, we observed consistent associations linking specific oral microbes involved in nitrate metabolism and cognitive function across both populations. These findings highlight that, while regional environmental and lifestyle factors could drive variation in oral microbiota and related physiological markers, there is growing evidence that key microbial and cognitive interactions may be robust and reproducible. Integrating geographical context will be essential to develop effective, tailored microbiome-based strategies that are broadly applicable across diverse populations and age groups.

### Significance statement

This study examined two independent cohorts from the UK and Belgium and found that, despite clear geographic differences in microbiome composition, age-related microbial patterns and their links to cognition were consistent. Older adults showed enrichment of *Treponema and Tannerella*, while younger adults showed enrichment of nitrate-reducing species such as *Neisseria flavescens*, which was associated with better cognitive performance in young adults. These findings demonstrate that geographic location differences may not obscure conserved microbial signatures related to cognitive ageing and provide a basis for future research investigating the potential mechanisms linking oral microbial composition with cognitive health.

## Supporting information

S1 Fig Principal component analysis (PCA) plots for young and older adults in Belgium and the UK based on Bray-Curtis. PCA plot Belgium (A) shows ordination of microbiome samples in young (blue) and older (red) adults. PCA plot UK (B) shows ordination of microbiome samples in young (blue) and older (red) adults. Arrows show the top ten taxa that contribute to the principal components. PERMANOVA of Bray-Curtis, *P* > 0.05.
(TIF)

S1 Table Age-dependent effects of plasma and salivary nitrate and nitrite concentrations on cognitive performance. Interaction analyses examining whether age modified the association between plasma or salivary nitrate and nitrite concentrations and cognitive performance in two independent cohorts. P-values were adjusted for multiple comparisons using the false discovery rate (FDR). No interaction terms reached statistical significance after FDR correction (q > 0.05).
(XLSX)
